# A Greek Nationwide Survey About Sources of Information on Seasonal Influenza and COVID-19 Vaccination Used by Healthcare Facility Staff During the Pandemic

**DOI:** 10.3390/healthcare13060670

**Published:** 2025-03-19

**Authors:** Ioanna Avakian, Katerina Dadouli, Stamatia Kokkali, Konstantinos Fotiadis, Christos Hadjichristodoulou, Varvara Α. Mouchtouri

**Affiliations:** 1 Laboratory of Hygiene and Epidemiology, Faculty of Medicine, University of Thessaly, 22 Papakyriazi Street, 41222 Larissa, Greece; joavakian@uth.gr (I.A.); adadouli@uth.gr (K.D.); stamatiakokkali@gmail.com (S.K.); xhatzi@uth.gr (C.H.); 2 Veria Hospital Unit, Hmathia General Hospital, 59132 Veria, Greece; kostasfotiad@yahoo.gr

**Keywords:** sources of information, COVID-19, seasonal influenza vaccination, healthcare workers, primary healthcare, vaccine hesitancy, vaccine acceptance

## Abstract

**Background**: Workers in healthcare facilities can encourage and serve as role models for the general population regarding vaccination. The information source preferences of employees in healthcare facilities can play an important role in their decisions to receive COVID-19 and seasonal influenza vaccinations (SIVs). A study of specific channels of information and their impact on vaccine acceptance could provide valuable insights. **Methods**: A cross-sectional questionnaire-based survey was conducted during the first semester of 2021 among 2592 staff members in healthcare facilities (primary, secondary and tertiary). **Results**: Higher odds of seasonal influenza vaccination (SIV) acceptance were found among staff who were informed by the National Public Health Organization (NPHO) (adjusted Odds Ratio (aOR): 1.47, 95% confidence intervals (CI): 1.13–1.90), the Hellenic Ministry of Health (HMH) (aOR: 1.50, 95% CI: 1.16–1.94) and the Healthcare Facilities Infection Control Committees (ICC) (aOR: 1.35, 95% CI: 1.06–1.73). Professionals who were more willing to accept a COVID-19 vaccine were more likely to obtain information from television (aOR: 1.43, 95% CI: 1.08–1.92), the ICC (aOR: 1.36, 95% CI: 1.03–1.81), the NPHO (aOR: 1.71, 95% CI: 1.28–2.28) and the HMH (aOR: 1.68, 95% CI: 1.26–2.26). Social media presented no statistically significant association with either COVID-19 vaccine acceptance or SIV. **Conclusions**: Workers in healthcare facilities who received information from highly credible organizations were more likely to accept vaccines. Television was effective in disseminating COVID-19 vaccine campaigns.

## 1. Introduction

The COVID-19 pandemic raised several issues regarding communicable diseases, including risk perception, preventive measures and information dissemination. COVID-19 vaccine hesitancy challenged the implementation of pandemic response strategies [1]. Sources of information used by both general and specific populations played a significant role in COVID-19 vaccination. Several studies have examined the association between vaccine acceptance and various sources of information, both formal and informal, to determine their impact on vaccination in different populations [2,3,4,5,6,7,8]. However, there is limited literature available on the preferences for information sources among workers in healthcare facilities and their associations with vaccination hesitancy [3].

Similarly, the seasonal influenza virus causes varying levels of acute respiratory disease severity, leading to increased hospitalization, mortality and morbidity, especially with populations such as infants, elderly people, pregnant women, patients with renal, cardiac or neurological conditions and Healthcare Workers (HCWs) [9,10]. Seasonal influenza was an additional risk factor during the COVID-19 pandemic, due to their shared transmission methods and symptoms [11,12]. HCWs may act as a reservoir of the virus, transmitting the disease to vulnerable patients [13].

One of the most effective interventions for preventing influenza epidemics is vaccination [14]. Although seasonal influenza vaccination (SIV) is a cost-effective and efficient practice, vaccination rates remain suboptimal in most regions [15]. Even though there are studies to suggest that the COVID-19 pandemic positively influenced SIV intention rates, vaccine hesitancy continues to hinder effective disease control [14].

Misconceptions and a lack of updated knowledge about vaccine safety and effectiveness could contribute to low rates of SIV [16,17]. On the other hand, HCWs’ awareness and knowledge regarding vaccination can lead to higher vaccine recommendation rates and uptake [18]. Pre-COVID-19 SIV increases the probability of COVID-19 vaccination, both in HCWs and the general population [8,19]. SIV also contributes to the maintenance of the functionality of the healthcare system as it reduces HCWs’ absenteeism from work and thus protects productivity and reduces overall costs [20,21,22].

Regarding the general population, those who received information from their healthcare providers were more likely to receive a vaccination [8]. On the contrary, evidence suggests that atypical sources of information, such as the social media, significantly contribute to the anti-vaccination movement [23]. Further, studies indicate that the COVID-19 vaccine acceptance rate can be influenced by both formal and informal sources including the internet, institutional sources, scientific literature and the opinions of others, both among the general public and HCW [24]. Restoring trust in traditional media such as television and promoting well-founded guidance on social media can improve access to high-quality information [25].

HCWs are an important source of information and guidance which the population trusts concerning vaccination, and they played a distinguished role in COVID-19 vaccination roll-out all over the world [26]. Even among colleagues, the encouragement of senior supervisors contributes to the vaccination of younger juniors [27]. Major concerns of the HCWs that lead to COVID-19 vaccine refusal are a lack of adequate information and vaccine safety issues [28,29,30,31,32,33].

A better understanding on how HCWs receive information may assist policymakers in implementing more effective training schemes and dissemination methods to raise vaccine coverage. In Greece, a number of studies examined the impact of information channels on HCWs’ decisions to receive COVID-19 vaccination [34,35]. Similar data for SIV remain limited [36]. The aim of the current study was to investigate whether the healthcare facilities workers’ intention to be vaccinated against COVID-19 and seasonal influenza in the first half of 2021 was influenced by the types of information sources used. Additionally, this study provides insights into specific sources of information in an individualized manner, an aspect that is scarcely explored in the existing literature, particularly in Greece.

## 2. Methods and Materials

### 2.1. Study Design

A cross-sectional questionnaire-based survey was designed and conducted from February to June 2021 in Primary (PHC), Secondary (SHC) and Tertiary healthcare facilities (THC) in Greece, in order to assess the knowledge, attitudes and practices of Rural and Urban Healthcare Centers (RHC/UHC) and Hospital (HO) personnel regarding COVID-19 pandemic and vaccination. The period when the survey took place coincided with the COVID-19 vaccination roll-out.

A total of 916 RHC/UHC personnel and 1045 HO workers were calculated using a Raosoft Digital Sample Size Calculator [37], applying 3% as a margin of error, 95% as the confidence interval (CI) and 50% as the expected frequency. The population size was 6456 and 50,000 for the RHC/UHC and HO professionals, respectively, according to the formal records of the country [38]. Moreover, the estimated response rate was 30%, relying on the distribution format and the workload of the personnel at the given period of time. The final sample size was approximately 3000 for RHC/UHC and 3500 for HO. A representative sample of 125 RHC/UHU and 20 HOs was selected, according to a geographically stratified sampling plan. All of the employees of the healthcare facilities were eligible for the study, including healthcare workers, administrative officers and other personnel.

The survey questionnaire included 25 questions and was developed by an expert team comprising academics, including a public health specialist, an epidemiologist and an occupational health professional. Guidelines published by WHO, advice issued by the Hellenic National Public Health Organization (NPHO) and the Ministry of Health, as well as relevant studies conducted in Greece, were the foundation for the development of the survey tool [34,39,40,41]. A pilot testing of the questionnaire was conducted in order to evaluate completion time, to appraise the clarity of questions and to test the online tool functionality.

The questionnaire featured inquiries about knowledge, attitudes and practices (KAP) concerning vaccinations overall, as well as SIV and COVID-19 vaccination specifically (Supplementary Material S1). We analyzed the responses to questions related to the sources of information that the target population used during the survey period in order to obtain information regarding the COVID-19 vaccine and SIV (see Supplementary Material S1—question 21). A variety of information sources were examined, including “general context publications” that represented publications other than newspapers, for example, lifestyle magazines, which were likely to promote alternative treatments or remedies for COVID-19 and SIV.

The questionnaire took approximately 15 min to complete. The processes for data collection, entry, analysis and storage adhered to the anonymity, privacy and confidentiality standards outlined by national legislation and the regulations of the University of Thessaly, Greece.

### 2.2. Internal Consistency Reliability

The internal consistency of the questionnaire was assessed using Cronbach’s alpha coefficient, which yielded a reliability score of 0.868, indicating strong internal consistency.

### 2.3. Ethical Statement

The survey received approval from the Ministry of Health and the Health District Authorities. Ethical approval was also granted by the scientific committee of the University of Thessaly (protocol numbers 49 and 48/13 January 2021). All participants gave written consent prior to completing the questionnaire and were thoroughly informed about the survey’s objectives and the regulations ensuring the protection of personal data (Supplementary Material S2).

### 2.4. Statistical Analysis

Statistical analyses were performed using the R programming language (R Core Team: R: A Language and Environment for Statistical Computing, Vienna, Austria: Foundation for Statistical Computing version 4.2.2) [42]. Continuous variables were reported as means ± standard deviations, while categorical variables were presented as frequencies and percentages. To determine optimal cut-off points for age and years of employment based on vaccination status, ROC analysis was employed. ROC curves were utilized to transform these continuous variables into categorical ones, facilitating the subsequent data analysis. The associations between outcomes and participants’ characteristics were evaluated using chi-square analysis or Student’s *t*-test. For continuous data, Student’s *t*-test was used, as there were no deviations from the normal distribution (verified by Shapiro–Wilk normality test) and no violations of the homogeneity of variance assumption (confirmed by Levene’s test). In the univariate analysis, dependent outcome percentages and prevalence ratios (PR) with 95% confidence intervals (CIs) were provided.

Stepwise bivariate logistic regression analysis with a 95% CI was employed to explore the direction of associations. Variables included in the bivariate logistic regression model were selected based on the previous literature. Specifically, the stepwise bivariate logistic regression model included demographic characteristics (Q1–Q11), personal or cohabitants’ medical history (Q12, Q13), knowledge-related questions (Q14, Q15 and Q22) and sources of information (Q21). All statistical tests were two-sided, and a *p*-value of <0.05 was considered statistically significant.

Three survey questions (Q14, Q15 and Q22) were scored on a five-point Likert scale: “completely disagree”, “disagree”, “neither agree nor disagree”, “agree” and “completely agree”. Responses of “completely disagree”, “disagree”, or “neither agree nor disagree” were categorized as disagreement, while responses of “completely agree” or “agree” were categorized as agreement. Each of the survey questions, Q14, Q15 and Q22 (Supplementary Material S1), contained three sub-items. Responses were considered correct if all three sub-questions were answered correctly; if at least one sub-question was answered incorrectly, the overall response was considered incorrect.

## 3. Results

### 3.1. Demographics

The total number of distributed questionnaires was 6500 and 2592 were completed and included in the survey sample (response rate 39.9%). A questionnaire was deemed eligible if it was fully completed. No missing data were identified.

The mean and median age of the study responders were 43.3 years and 44.0 years, respectively. Most of the participants were female (70.9%) and married (63.3%). Several of the respondents attained a higher-level degree (higher educational institution/university, 30.9%) or a master’s/doctorate (24.3%). Physicians and nurses comprised 38.1% and 39.0% of the sample, respectively. The vast majority of participants were parents (59.4%) and worked in secondary healthcare facilities (56.2%), while the median years of practice was 15.4 (IQR: 10.7). The proportions of answers regarding the sources of information used—television, social media, general context publications and general context websites—were 48.1, 40%, 26.7% and 36.2%, respectively.

### 3.2. Univariate and Multivariate Analysis

Table 1 demonstrates association between sources of information and vaccination for seasonal influenza, COVID-19 and both diseases. Generally, univariate analysis reveals that formal sources of information have a positive statistically significant association with all kinds of vaccination. In addition, information from social media and general context websites was negatively associated with the SIV and no statistically significant association with COVID-19 vaccination was detected.

A further descriptive analysis is represented in Table 2, where associations of each source of information with demographic characteristics are presented.

The association of sources of information and the correct answers in the knowledge (Q14) and attitude (Q15) questions was examined in a previous paper [19]. Moreover, the association between employment and the correct answers in the knowledge (Q14) and attitude (Q15) questions was explored (Supplementary Material S4). According to the univariate analysis, the health promotion specialists had higher odds to answer the knowledge questions correctly in comparison to the physicians (PR: 1.31, 95% CI: 1.09–3.53), while all other professional groups had lower odds. In addition, concerning the attitude questions (Q15), all of the workers’ groups had lower odds of answering correctly compared to the physicians (Supplementary Material S4). Regarding the auto-perception of the level of information (Q20) and its association with the questions concerning knowledge (Q14) and attitudes (Q15), the higher the amount of information stated by the participants, the higher the percentage of correct answers in the given questions (Supplementary Material S5).

Stepwise binary logistic regression (Table 3) concerning SIV showed positive statistically significant associations with ICC (aOR: 1.35, 95% CI: 1.06–1.73), NPHO (aOR: 1.47, 95% CI: 1.13–1.90) and the website of the HMH (aOR: 1.50, 95% CI: 1.16–1.94). Regarding COVID-19 vaccination acceptance, positive statistically significant associations were present with the television (aOR: 1.43, 95% CI: 1.08–1.92), the ICC (aOR: 1.36, 95% CI: 1.03–1.81), the NPHO (aOR: 1.71, 95% CI: 1.28–2.28) and the HMH (aOR: 1.68, 95% CI: 1.26–2.26) as sources of information.

## 4. Discussion

This study investigates the impact of channels that the personnel of healthcare facilities used to draw information from during the pandemic in order to make their decision on SIV and COVID-19 vaccination. The ICC, the NPHO and the HMH were regarded as formal sources of information. The SIV and COVID-19 vaccination coverage was 69.4% and 81.1%, respectively, and the related data have been published in previous papers [19,29,30].

According to our study, a vast proportion of the participants, despite the fact that they work in the facilities of primary, secondary and tertiary sectors of healthcare, obtained information from sources other than those that are formal or considered scientifically valid, such as social media, television and general context websites and publications. In the literature, limited data are available on the proportion of HCWs who obtained information from informal channels such as social media and television. Further research on how unconventional sources of information could support public health initiatives and relevant intervention projects might be of great value.

Our survey suggests that professionals who prefer institutional sources of information are more accepting to both SIV and COVID-19 vaccination. Our results are comparable with those of other studies, although the methods and tools of investigation differ largely from study to study [3,34,35,43,44,45,46].

An intriguing finding from our survey is that television played a significant role in shaping attitudes toward COVID-19 vaccines. Participants who used television to stay informed during the study period were more receptive to COVID-19 vaccination. During the pandemic in Greece, television was the main means of information that authorities used to reach citizens. The NPHO, HMH and expert group messages were broadcast on a daily basis and constantly in order to provide updates to the population and to encourage their physical and mental wellbeing. In this context, the above mentioned association between television and COVID-19 vaccination can be expected. There were no similar strategies for SIV.

Concerning social media, our study revealed that its impact on the decision of healthcare facilities workers for COVID-19 and SIV was not statistically significant. However, several studies identified a negative association between vaccination acceptance and the use of social media as an information source [35,44,45,47]. A possible explanation could be when the survey period occurred and the target population. It is possible that healthcare facility personnel are more likely to obtain information from trusted organizations via social media and participate in knowledgeable social networks that are familiar with health issues and are more accepting to lifestyles that adhere to Public Health Authorities guidance.

The survey results provide useful insights for stakeholders regarding the management of public health campaigns using different sources of information and the importance of the target population. In addition, television has been proven to be an efficient channel of information in order to disseminate scientific knowledge and good public health practices in Greece

Our study has limitations. As a cross-sectional study, it only captured a snapshot of a particular time, making it difficult to infer causal relationships between information sources and vaccination behavior. A longitudinal study could provide stronger evidence on how information sources influence decision-making over time. The sample of the study is convenient, and, thus, the generalization of the results in the target population is limited and participation bias could have occurred; hesitant respondents could be less likely to participate as their attitude was different from the expert’s advice. However, in a previously published paper of the same research topic regarding SIV and COVID-19 vaccine acceptance, the observed vaccination rate was comparable with the rate of the Greek National Registry for COVID-19: 81.1% (77.7–85.3%) and SIV: 69.4% [19,29,30]. Due to the convenient sample, the representativeness in terms of demographics and comparing to the total population of primary, secondary and tertiary healthcare facilities personnel is limited and not well established. Unfortunately, no formal data regarding healthcare personnel population demographics were available during the period of the survey. In addition, the response rate of the study could pose a limitation, but, considering the mixed method of questionnaire distribution, the level of response rate may be considered acceptable [48]. Another disadvantage of our study is the fact that the survey questionnaire did not include sources of information related to a doctor’s consultation or friend and family advice. Although these channels of information are frequently mentioned in the literature, the researchers decided to keep the length of the questionnaire limited in order to facilitate the participation of the target professionals, who at the given period were dealing with an enormous workload. Furthermore, our study did not collect information on political beliefs, workplace culture, or previous vaccine experiences, which are factors that could potentially influence vaccine hesitancy.

## 5. Conclusions

The impact of sources of information on healthcare personnel’s SIV and COVID-19 vaccination is significant and should be taken into consideration during public health policy development. Although healthcare personnel are expected to rely on scientific channels of guidance, advice from unconventional sources might influence their vaccination decision. However, disseminating scientific knowledge through television could be an effective way to reach healthcare facilities professionals in the context of good public health practice.

## Figures and Tables

**Table 1:** Association of sources of information and seasonal influenza vaccination (SIV) and COVID-19 vaccination through univariate analysis.

Sources of Information (Always/Often vs. Rarely/Never)	SIV (Vaccinated vs. Not-Vaccinated) PR, 95% CI	COVID-19 (Vaccinated vs. Not-Vaccinated) PR, 95% CI	SIV + COVID-19 (Vaccinated vs. Not-Vaccinated) PR, 95% CI
TV	0.94 (0.89–0.99)	0.97 (0.94–1.01)	0.98 (0.96–1.01)
Social media	0.92 (0.87–0.97)	0.98 (0.94–1.02)	0.98 (0.96–1.01)
Newspaper (print or electronic versions)	1.09 (1.03–1.14)	1.05 (1.01–1.09)	1.04 (1.01–1.07)
General context publications	1.02 (0.96–1.08)	1.03 (0.99–1.07)	1.02 (0.99–1.05)
Articles in medical journals	1.32 (1.24–1.41)	1.18 (1.13–1.24)	1.13 (1.09–1.17)
Infection Control Committee at health facility (ICC)	1.15 (1.09–1.21)	1.09 (1.05–1.13)	1.09 (1.05–1.12)
General context websites	0.93 (0.88–0.99)	1.02 (0.98–1.06)	1.00 (0.97–1.03)
Hellenic National Public Health Organization (NPHO)	1.28 (1.20–1.36)	1.17 (1.11–1.22)	1.12 (1.08–1.16)
Website of the Hellenic Ministry of Health (HMH)	1.25 (1.18–1.32)	1.14 (1.10–1.19)	1.11 (1.07–1.14)

PR: prevalence ratio; CI: confidence interval.

**Table 2:** Association of sources of information and demographic characteristics—univariate analysis.

	Sources of Information PR, 95% CI (Always/Often vs. Rarely/Never)								
Demographic Characteristics	TV	Social Media	Newspaper	General Context Publications	Medical Articles	ICC	General Interest Websites	NHPO	HMH
Age									
≤53 vs. >53 (years)	0.86 (0.78–0.94)	1.09 (0.97–1.23)	1.07 (0.94–1.22)	1.04 (0.88–1.24)	1.23 (1.13–1.34)	0.92 (0.84–1.01)	1.16 (1.00–1.34)	1.08 (1.00–1.17)	1.08 (0.99–1.20)
Gender									
Male vs. female	0.89 (0.81–0.98)	0.89 (0.81–0.99)	1.04 (0.94–1.16)	1.05 (0.91–1.21)	1.07 (1.01–1.13)	0.96 (0.89–1.05)	0.86 (0.76–0.97)	0.86 (0.80–0.92)	0.80 (0.73–0.87)
Marital Status									
Married vs. non-married	1.13 (1.04–1.23)	1.03 (0.95–1.13)	1.13 (1.02–1.25)	1.02 (0.89–1.16)	0.92 (0.87–0.98)	1.06 (0.98–1.14)	1.15 (1.03–1.28)	1 (0.95–1.06)	1.06 (0.98–1.15)
Education Level									
Second grade vs. third grade education	1.31 (1.16–1.48)	1.09 (0.94–1.29)	0.71 (0.56–0.90)	0.62 (0.44–0.86)	0.49 (0.40–0.60)	0.68 (0.56–0.83)	1.07 (0.88–1.30)	0.61 (0.51–0.73)	0.61 (0.49–0.76)
Profession									
Physician vs. non-physician	0.65 (0.60–0.72)	0.77 (0.70–0.85)	1.06 (0.97–1.17)	0.97 (0.85–1.11)	1.60 (1.52–1.68)	1.16 (1.08–1.25)	0.74 (0.66–0.83)	1.22 (1.16–1.29)	1.17 (1.09–1.27)
Sector of Employment									
Primary healthcare vs. secondary healthcare	0.80 (0.75–0.88)	0.95 (0.87–1.04)	1.14 (1.04–1.25)	0.86 (0.76–0.98)	1.19 (1.13–1.26)	0.95 (0.88–1.02)	0.93 (0.84–1.03)	1.37 (1.30–1.44)	1.61 (1.49–1.74)
District of Profession									
3rd, 4th, 5th vs. 1st, 2nd, 6th, 7th health district	1.18 (1.09–1.29)	1.06 (0.97–1.15)	0.93 (0.85–1.03)	0.98 (0.86–1.11)	0.83 (0.78–0.87)	0.91 (0.84–0.98)	0.95 (0.86–1.06)	0.92 (0.87–0.97)	0.88 (0.81–0.94)
Years of Employment									
≤21.5 vs. >21.5 (years)	0.80 (0.73–0.86)	1.01 (0.92–1.11)	1.01 (0.91–1.12)	0.96 (0.83–1.10)	1.13 (1.06–1.20)	0.91 (0.84–0.98)	0.98 (0.90–1.08)	1.05 (0.98–1.11)	1.01 (0.93–1.10)

PR: prevalence ratio, CI: confidence interval.

**Table 3:** Stepwise binary logistic regression: association between sources of information and SIV/COVID-19 vaccination.

Sources of Information Always/Often vs. Rarely/Never	SIV (Vaccinated vs. Not-Vaccinated) aOR wth 95% CI	COVID-19 (Vaccinated vs. Not-Vaccinated) aOR wth 95% CI
Television	excluded variable	1.43 (1.08–1.92)
Social media	0.81 (0.64–1.04)	1.23 (0.93–1.63)
Newspaper	1.21 (0.94–1.55)	excluded variable
General context publications	excluded variable	excluded variable
Articles in medical journals	1.25 (0.96–1.63)	excluded variable
Infection control committee at health facility (ICC)	1.35 (1.06–1.73)	1.36 (1.03–1.81)
General interest websites	excluded variable	1.35 (1.00–1.81)
Hellenic National Public Health Organization (NPHO)	1.47 (1.13–1.90)	1.71 (1.28–2.28)
Website of the Hellenic Ministry of Health (HMH)	1.50 (1.16–1.94)	1.68 (1.26–2.26)

aOR: adjusted Odds Ratio; CI: confidence interval adjusted with demographics q1–11, q12, q13, q14, q15, q22 (Supplementary Material S1). Excluded variables: These variables were considered in the stepwise selection process but did not meet the criteria (e.g., statistical significance or contribution to model fit) to be retained in the model.

## Data Availability

The dataset is available on request from the authors.

## Supplementary Materials

The following supporting information can be downloaded at https://www.mdpi.com/article/10.3390/healthcare13060670/s1: Material S1: Survey Questionnaire. Material S2: Informed Consent. Material S3: Descriptives. Material S4: Univariate analysis between knowledge (Q14)/attitudes (Q15) and employment. Material S5: Association of Knowledge (Q14)/attitudes (Q15) questions with Level of information (Q20).
