# Supplementary material for: A Greek Nationwide Survey About Sources of Information on Seasonal Influenza and COVID-19 Vaccination Used by Healthcare Facility Staff During the Pandemic
Source: Healthcare (Basel). 2025 Mar 19;13(6):670. doi: 10.3390/healthcare13060670 (PMC11941847; doi:10.3390/healthcare13060670)
Supplement: Supplementary file 1 [file healthcare-13-00670-s001.zip › healthcare-3480929-supplementary.pdf]

Supplementary material

S1 : Survey Questionnaire

UNIVERSITY OF THESSALY
SCHOOL OF HEALTH SCIENCES
FACULTY OF MEDICINE
DEPARTMENT OF HYGIENE & EPIDEMIOLOGY

QUESTIONNAIRE ON KNOWLEDGE, ATTITUDES AND PRACTICES OF HEALTH CARE PROFESSIONALS RELATED TO THE SARS-CoV-2 VACCINE

GENERAL SECTION

1. Age: …….. years
2. Gender: Male Female
3. Marital status (indicate with 'X'): Married Unmarried Other (please specify)……………

4. Education level (indicate with 'X'):
High school Institute of Vocational Training (IEK)
Technological Educational Institute (TEI) Higher Education Institute/University (AEI)
Master /Doctoral

5. Health care profession (indicate with 'X'):
Doctor (please indicate specialization ……………………………) Nurse
Medical Laboratory worker Other (please specify)..............................

6. Sector of employment (indicate with 'X'):
Private hospital Public hospital
Health centre (K.Y.) Community-based primary health unit (To. M.Y)

7.Health District (Υ.ΠΕ) of employment: ………………………………………………

8. Regional Unit of employment: …………………………………………..

9. Department of employment (indicate with 'X'): (please do not respond if you work at a health centre or community-based primary health unit)
Clinical Laboratory Other (please specify)..............................

10. Section of employment (indicate with 'X'): (please do not respond if you work at a health centre or community-based primary health unit)
Pathology Surgery
Laboratory Other (please specify)..............................

11. Years of practice: ....…………

PART A
12. Do you belong to a vulnerable/high risk group due to your medical history? (indicate with 'X'):
(cardiovascular disease, respiratory disease, diabetes, immunosuppression, cancer, pregnancy etc.)
YES NO

13. Do you live with older individuals or individuals belonging to a vulnerable/high risk group due to their medical history? (indicate with 'X'):
(cardiovascular disease, respiratory disease, diabetes, immunosuppression, cancer, pregnancy etc.)
YES NO

14. Please circle the choice that best indicates your response.

	Completely agree	Agree	Neither agree nor disagree	Disagree	Completely disagree
The HPV vaccine is recommended for all males up to 18 years of age in the country.	1	2	**3**	4	5
After the flu vaccination, certain foods are not permitted to be consumed for a period of 24 hours.	1	2	**3**	4	5
One of the contraindications of the flu vaccine is an allergy to eggs.	1	2	**3**	4	5

15. Please circle the choice that best indicates your response.

	Completely agree	Agree	Neither agree nor disagree	Disagree	Completely disagree
Vaccinations are an important tool for the protection of public health and in particular of health professionals and workers in the health sector	1	2	**3**	4	5
Natural immunity acquired via disease is always preferable to immunity acquired via vaccination.	1	2	**3**	4	5
Many vaccines often have serious side effects.	1	2	**3**	4	5

16. Are you the parent/guardian of one or more children? (indicate with 'X'):
YES NO
If **YES,** do you adhere to the child vaccination program suggested by the National Vaccination Program in the country? (please circle the answer of your choice)
YES, I vaccinate my children according to the National Vaccination Program
I select and carry out some vaccinations I do not vaccinate my children

17. Have you been vaccinated with the seasonal flu vaccine? (indicate with 'X'):

YES NO

If not, please indicate why (more than one response can be selected)
I do not have time
Apathetic
I use homeopathic remedies
I do not think I am at risk
Fear regarding vaccine safety
Other (please specify)..............................

PART B

18. Do you know of a relative or friend who has had COVID-19? (indicate with 'X'):

YES NO

19. Do you come into contact with COVID-19 patients while performing your job duties? (indicate with 'X'):
YES NO

20. How do you evaluate your level of being informed about vaccines against the SARS-CoV-2 virus that causes COVID-19? (Please circle below the option which best represents your answer)

No information	Insufficient	Satisfactory	Excellent
1	2	3	4

21. Which channels do you use to keep informed about the COVID-19 pandemic and the SARS-CoV-2 vaccine, and how often? (please circle the option that represents your answer)

	Always	Often	Rarely	Never
Television	1	2	3	4
Social media channels (Facebook, Twitter, Instagram etc.)	1	2	3	4
Newspaper (in print or electronic versions)	1	2	3	4
General context publications/journals (in print or electronic versions)	1	2	3	4
Medical articles in journals (in print or electronic versions)	1	2	3	4
Committee for infectious diseases at health facility	1	2	3	4
General context websites	1	2	3	4
Website of the Hellenic National Public Health Organization (NPHO)	1	2	3	4
Website of the Hellenic Ministry of Health	1	2	3	4

22. Please circle the response below that represents your answer.

	**Completely agree**	**Agree**	**Neither agree nor disagree**	**Disagree**	**Completely disagree**
Some of the vaccines against SARS-CoV-2 which are approved and used in the country are based on mRNA technology	1	2	3	4	5
The dosage regimen of the vaccines against SARS-CoV-2 includes 3 doses	1	2	3	4	5
There is evidence that mRNA technology interferes with the DNA of cells	1	2	3	4	5

23. Have you been or will you be vaccinated with any of the vaccines against the SARS-CoV-2 virus which causes COVID-19, which have received the necessary approvals from the European Medicines Agency and the National Medicines Agency? (indicate with 'X'):

YES NO

If not, please indicate why (**indicate with 'X'**) (more than one response can be selected)

- I do not have time
- Apathetic
- Fear regarding vaccine safety
- I do not think I am at risk
- I need further information in order to make a decision

Other (please specify) ………………………………….……………………………………………………

24. Does the short period of time for development of the vaccines cause you any concerns about its safety? (circle the option below that represents your answer)

**Completely agree**	**Agree**	**Neither agree nor disagree**	**Disagree**	**Completely disagree**
**1**	**2**	**3**	**4**	**5**

25. Do you believe that vaccination against SARS-CoV-2 should be mandatory for healthcare professionals? (indicate with 'X'):

YES NO

THANK YOU FOR YOUR TIME

S2 Informed Consent

UNIVERSITY OF THESSALY
SCHOOL OF HEALTH SCIENCES
FACULTY OF MEDICINE
DEPARTMENT OF HYGIENE & EPIDEMIOLOGY

Informed Consent

The signatory ………………………………………………………………………………………

I agree to fill in the anonymous questionnaire called ***QUESTIONNAIRE ON KNOWLEDGE, ATTITUDES AND PRACTICES RELATED TO SEASONAL INFLUENZA VACCINATION AND THE SARS-CoV-2 VACCINE*** and take part in survey of the Department of Hygiene and Epidemiology of Faculty of Medicine of the University of Thessaly.

I declare that I am informed from the researchers about the aim of the research. The collected data will only be used for the aim of survey and all personal information will be protected, according to GDPR. All data and results of the study belong to the University of Thessaly, according the Code of Conduct of UTH

This study has been approved by the Ethics Committee of University of Thessaly [Protocol Number. 49/ 13.01.2021 and 48/ 13.01.2021]. For more information about the program, participants can contact the following researchers Mr Fotiadi Konstantino(tel 6906727581,mail: kostasfotiad@yahoo.gr) and Ms Avakian Ioanna(tel 6948381262 , mail: joavakian@med.uth.gr). For any complaints or legal charges about the research's prosecution participants can contact the Ethics Committee of University of Thessaly (ehde@uth.gr). For charges regarding the management of personal data participants can contact the Head of Personal Data Management of University of Thessaly(dpo@uth.gr) and in any case the Data Protection Supervisor. (complaints@dpa.gr)

Signature:

Christos Hadjichristodoulou

Professor of Hygiene and Epidemiology
Director of the Department of Hygiene and Epidemiology
University of Thessaly

S3 Descriptives

Variables		N(%)
Age (binary)	≤53	2138 (82.5%)
	>53	451 (17.4%)
	Missing	3 (0.1%)
Gender	Male	754 (29.1%)
	Female	1838 (70.9%)
Marital Status	NA	55 (2.1%)
	Divorced	95 (3.7%)
	Widowed	7 (0.3%)
	Unmarried	794 (30.6%)
	Married	1641 (63.3%)
Education level	High School	177 (6.8%)
	IEK	216 (8.3%)
	TEI	767 (29.6%)
	MSc/PhD	631 (24.3%)
	BSc	800 (30.9%)
	Missing	1 (0.0%)
Profession	Physician	987 (38.1%)
	administrative	178 (6.9%)
	Medical Laboratory worker	85 (3.3%)
	Midwife	55 (2.1%)
	Health professionals providing consultation	50 (1.9%)
	Other health professionals	170 (6.6%)
	Nurse	1010 (39.0%)
	Health promotion specialist	57 (2.2%)
Sector of employment	Private hospital	12 (0.5%)
	ToMY	153 (5.9%)
	Health Center	983 (37.9%)
	Public health	1444 (55.7%)
District of profession	1	189 (7.3%)
	2	214 (8.3%)
	3	442 (17.1%)
	4	512 (19.8%)
	5	602 (23.2%)
	6	385 (14.9%)
	7	248 (9.6%)
District of profession (binary)	1,2,6,7	1036 (40.0%)
	3,4,5	1556 (60.0%)

**Years of employment (binary)**	≤21.5	1831 (70.6%)
	>21.5	738 (28.5%)
	Missing	23 (0.9%)
**Sources of information Always/often**	TV	1243 (48.1%)
	Social media	1150 (40.0%)
	Newspaper (print or electronic versions)	1032 (44.5%)
	General context publications	689 (26.7%)
	Medical articles in journals	1731 (67.1%)
	Infection Control Committee at health facility (ICC)	1345 (52.2%)
	General context websites	932 (36.2%)
	Hellenic National Public Health Organization (NPHO)	1724 (66.8%)
	Website of the Hellenic Ministry of Health (HMH)	1308 (50.7%)

N: number of observations,

S4 Univariate analysis between knowledge(Q14) / attitudes(Q15) and employment

Employment	Q14 (correct vs incorrect)<br>PR, 95%CI	Q15 (correct vs incorrect)<br>PR, 95%CI
Physician	Ref	
administrative	0.69 (0.56-0.85)	0.29 (0.21-0.40)
Medical Laboratory worker	0.16 (0.08-0.32)	0.18 (0.09-0.33)
Midwife	0.80 (0.59-1.10)	0.48 (0.32-0.73)
Health consultant	1.19 (0.95-1.49)	0.83 (0.47-1.47)
Other health professionals	0.60 (0.48-0.75)	0.40 (0.30-0.52)
Nurse	0.48 (0.42-0.54)	0.34 (0.29-0.38)
Health promotion specialist	1.31 (1.09-3.53)	0.72 (0.54-0.98)

PR: prevalence ratio, CI : Confidence intervals

S5. Association of Knowledge (Q14) / attitudes (Q15) questions with Level of information (Q20)

Q20: Level of information	No information (N=39)	Insufficient (N=399)	Satisfactory (N=875)	Excellent (N=181)	P-value
**Q14**					
Correct	7 (17.9%)	110 (27.6%)	384 (43.9%)	89 (49.2%)	<0.001
Incorrect	32 (82.1%)	289 (72.4%)	491 (56.1%)	92 (50.8%)	
**Q15**					
Correct	3 (7.7%)	71 (17.8%)	317 (36.2%)	99 (54.7%)	<0.001
Incorrect	36 (92.3%)	328 (82.2%)	558 (63.8%)	82 (45.3%)	
